# PD-1 and PD-L1 are more highly expressed in high-grade bladder cancer than in low-grade cases: PD-L1 might function as a mediator of stage progression in bladder cancer

**DOI:** 10.1186/s12894-018-0414-8

**Published:** 2018-11-06

**Authors:** Takashi Kawahara, Yukari Ishiguro, Shinji Ohtake, Ikuma Kato, Yusuke Ito, Hiroki Ito, Kazuhide Makiyama, Keiichi Kondo, Yasuhide Miyoshi, Yasushi Yumura, Narihiko Hayashi, Hisashi Hasumi, Kimito Osaka, Kentaro Muraoka, Koji Izumi, Jun-ichi Teranishi, Hiroji Uemura, Masahiro Yao, Noboru Nakaigawa

**Affiliations:** 1 Department of Urology, Yokohama City University Graduate School of Medicine, 3-9 Fukuura, Kanazawa-ku, Yokohama, Kanagawa 2360004 Japan; 2 Department of Pathology, Yokohama City University Hospital, Yokohama, Japan; 3 Department of Urology and Renal Transplantation, Yokohama City University Medical Center, Yokohama, Japan

**Keywords:** Programmed cell death protein 1, Programmed death-ligand 1, B7-H1, CD274, STAT1, NFATc1

## Abstract

**Background:**

Bladder cancers have been characterized as a tumor group in which the immunological response is relatively well preserved. Programmed death ligand 1 (PD-L1, B7-H1, CD274) has been shown to be expressed in several malignancies, including bladder cancer. However, the clinicopathological impact of this biomarker has not yet been established. In the present study, a quantitative real-time polymerase chain reaction (qPCR) was performed using paired normal and cancerous bladder cancer tissue to investigate *PD-1*/*PD-L1* gene expression.

**Methods:**

We examined the mRNA expression of *PD-1*/*PD-L1* by a qPCR using 58 pairs of normal and cancerous human bladder tissue specimens. We also examined the correlation with the expressions of the *STAT1* and *NFAT* genes, which are thought to be upstream and downstream of the *PD-L1* pathway, respectively.

**Results:**

There were no significant differences between normal and cancerous tissue in the expression of the *PD-1* and *PD-L1* genes (*p* = 0.724 and *p* = 0.102, respectively). However, *PD-1* and *PD-L1* were both more highly expressed in high-grade bladder cancer than in low-grade bladder cancer (*p* < 0.050 and *p* < 0.010). *PD-L1* was positively correlated with the expressions of both the STAT1 (*r* = 0.681, *p* < 0.001) and the *NFATc1* genes (*r* = 0.444. p < 0.001).

**Conclusions:**

*PD-1* and *PD-L1* might be a new biomarker that correlates with the pathological grade of bladder cancer. *PD-L1* might function as a mediator of stage progression in bladder cancer and S*TAT1*-*NFAT* pathway might associate this function.

**Electronic supplementary material:**

The online version of this article (10.1186/s12894-018-0414-8) contains supplementary material, which is available to authorized users.

## Background

Bladder cancer has been characterized as a tumor group in which the immunological response is relatively well preserved [1–4]. Programmed death ligand 1 (PD-L1, B7-H1, CD274) is expressed in several malignancies, including bladder cancer [5–7]. The clinicopathological impact of this biomarker has not been established across different tumor types [8]. The programed death-1 (PD-1)/PD-L1 pathway negatively regulates T cell activation and has been suggested to play an important role in regulating host antitumor immunity [1].

Recently, several clinical trials targeting PD-1/PD-L1 pathway using anti-PD-1 antibody or anti-PD-L1 antibody demonstrated the obvious benefit for the patients with urothelial cancer and were approved by Food and Drug Administration in the United States [9–11]. Interestingly, the anti-tumor effect of atezolizumab, the anti-PD-L1 antibody, was dependent on the PD-L1 expression status on tumor-infiltrating immune cells [9]. On the other hand, the anti-tumor effect of pembrolizumab, an anti-PD-1 antibody, was not affected by the PD-L1 expression in the tumor and infiltrating immune cells [10], nor was the anti-tumor effect of nivolumab (also an anti-PD-1 antibody) affected by the PD-L1 expression in tumor cells [11]. In addition, some investigators reported that bladder cancer expressing high PD-L1 showed a poor prognosis [1, 12, 13], but others suggested high PD-L1 predicted the good prognosis [14]. Thus, the correlation between *PD-L1* expression and the prognosis remains controversial.

The upregulation of *PD-L1* expression by tumor cells, however, is thought to be a mechanism by which solid tumors develop a tolerance to immune regulation [15]; however, the detailed mechanism of PD-L1 in urothelial carcinoma remains unknown.

We previously reported the anti-cancer progression role in signal transducer and activator of transcription 1 *(STAT1)* and nuclear factor of activated T-cells *(NFAT)* [16–18]. This study evaluated the expressions of both *PD-1* and *PD-L1* in urothelial carcinoma and firstly examined the *PD-L1* related genes: *STAT1* and *NFAT*.

## Methods

### Patients and tissue specimens

To analyze gene expression, we retrieved 58 pairs (116 specimens) of bladder tissue specimens that were obtained via transurethral resection of the bladder at Yokohama City University Hospital (Yokohama, Japan) and frozen immediately after resection. Appropriate approval was obtained from the institutional review board at our institution. The median age of the patients at resection was 72 years (mean age: 70.2 ± 12.6 years). All patients were histologically diagnosed with bladder cancer, including 22 (37.9%) patients who were diagnosed with low-grade bladder cancer and 21 (36.2%) patients who were diagnosed with high-grade bladder cancer (Table 1). Both cancerous and non-cancerous tissue was pathologically obtained, and the non-cancerous tissue was obtained at least 1 cm away from the tumor site. The median follow-up period was 2.8 years. None of the patients received any pre-operative therapies, including BCG, radiation, or other anticancer drugs.

**Table 1:** Patients’ Background

	Median (mean ± SD) or n (%)
No. of Pts.	58
Age (yr)	72 (70.2 ± 12.6)
Male / Female	10 (17.2%) / 48 (82.8%)
Pathological Grade	
Low	22 (37.9%)
High	21 (36.2%)
unknown / other	15 (25.9%)
Pathological Stage	
Ta	23 (39.7%)
> T1	19 (32.8%)
unknown	16 (27.6%)
Death (n, %)	5 (6.9%)

### Cell lines and Western blotting

Human urothelial carcinoma cell lines (UMUC3, TCC-SUP HTB-3, T24, and 5637) obtained from the American Type Culture Collection (Manassas, VA, USA) were maintained in Dulbecco’s modified Eagle’s medium (DMEM; Mediatech, Manassas, VA, USA) supplemented containing 10% fetal bovine serum (FBS) with penicillin (100 units/mL) and streptomycin (100 units/mL) at 37 °C in a humidified atmosphere of 5% CO_2_. Protein extraction and Western blotting were performed as described previously with minor modifications. In brief, equal amounts of protein (30–50 μg) obtained from cell extracts were harvested for a total protein analysis. Extracted protein was separated using 10% sodium dodecylsulfate (SDS)-polyacrylamide gel electrophoresis (PAGE) and transferred to a polyvinylidene difluoride membrane (Immun-Blot PVDF Membrane; BIO-RAD, Hercules, CA, USA) by electroblotting using a standard protocol.

### Quantitative real-time RT-PCR

Total RNA (0.5 μg), which was isolated from the bladder tissue specimens, using Isogen (NipponGene, Tokyo, Japan), was reverse transcribed using 1 μM oligo (dT) primers (Qiagen, Germantown, MD, USA) and 4 units of Omniscript reverse transcriptase (Qiagen, Germantown, MD, USA) in a total volume of 20 μL. Real-time quantitative PCR (qPCR) was then performed (StepOne Real Time PCR System, Applied Biosystems, Grand Island, NY, USA), using Fast SYBR Green Mastermix (Applied Biosystems, Grand Island, NY, USA), as described previously (22086872) [19] The following primer pairs were used for the RT-PCR: human *PD-L1*: forward 5′- CCA AGG CGC AGA TCA AAG AGA’; reverse 5′- AGG ACC CAG ACT AGC AGC A -3′, and human *NFATc1*: forward 5′- GTC CCA CCA CCG AGC CCA CTA CG -3′; reverse 5′- GAC CAT CTT CTT CCC GCCC ACG AC -3′. Human *GAPDH*: forward 5’-CTC CTC CAC CTT TGA CGC TG-3′; reverse, 5’-CAT ACC AGG AAA TGA GCT TGA CAA-3′ was used as an internal control. The sequences of these primers were acquired from the Primer Bank: 19906719 [20], 19,108,745 [21], 14,654,707 [22]. The *PD-1* and *STAT1* gene expressions were determined using TaqMan® Gene Expression Assays (*PD-L1* and *STAT1*, Applied Biosystems, Grand Island, NY, USA). All of the specific expression levels were divided by the quantity of *GAPDH* that was used.

### Statistical analyses

The patients’ characteristics were analyzed by the Mann-Whitney *U* and chi-square tests. Continuous variables are expressed as the median and as the mean (±SD). The patients’ survival rates were calculated by the Kaplan-Meier method and comparisons were made by a log-rank test. A *P* value of < 0.05 was considered to indicate statistical significance. A statistical analysis was performed and figures were created using the Graph Pad Prism software program (Graph Pad Software, La Jolla, CA, USA).

## Results

### PD-L1 expression was observed in bladder cancer cell lines

*PD-L1* was expressed in all human bladder cancer cell lines according to Western blotting (Additional file 1: Figure S1).

### PD-1 and PD-L1 are more highly expressed in high-grade bladder cancer than in low-grade bladder cancer

**Fig. 1:**
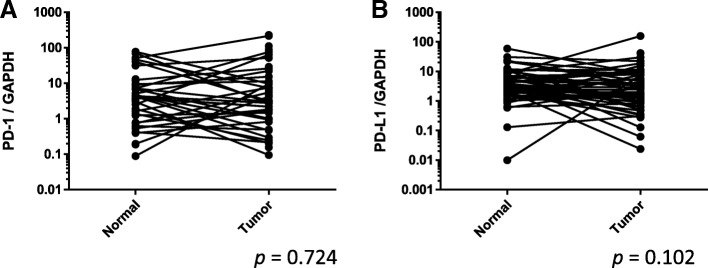
PD-1 and PD-L1 gene expression in paired normal and tumor tissue specimens

**Fig. 2:**
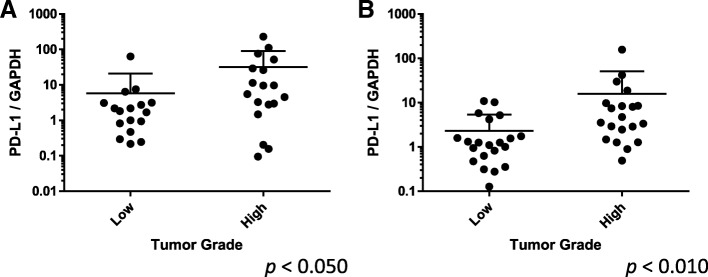
PD-1 and PD-L1 gene expression with consideration of pathological grade

*PD-1* and *PD-L1* were expressed in human bladder cancer tissues. There were no differences between normal and cancerous bladder tissue both *PD-1* and *PD-L1* gene expression (*p* = 0.724, *p* = 0.102, respectively) (Fig. 1). Both *PD-1* and *PD-L1* showed higher expression in high-grade tumors than in low-grade tumors (*p* < 0.050, *p* < 0.010, respectively) (Fig. 2). No correlation was found with the overall survival or pathological T stage.

### PD-L1 expression was correlated with STAT1 and NFATc1 expression

**Fig. 3:**
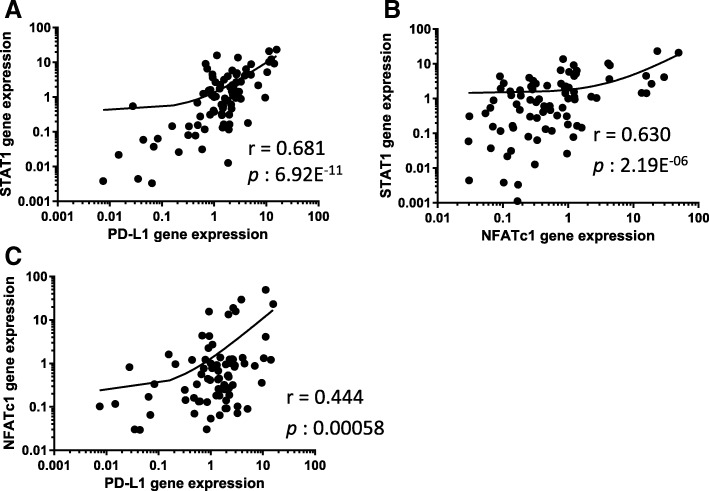
The correlations among PD-L1, STAT1, and NFATc1 gene expression

To compare the expression of the *PD-L1* gene, we analyzed the expressions of the *STAT1 and NFATc1* genes in addition to the expression of *PD-1*. The expressions of *PD-1* and *PD-L1* were positively correlated, but not strongly (*r* = 0.224, *p* < 0.05). *PD-L1* was also positively correlated with *STAT1* (*r* = 0.681, *p* < 0.001) and *NFATc1* (*r* = 0.444. *p* < 0.001) (Fig. 3).

## Discussion

This study demonstrated that *PD-L1* was more highly expressed in high-risk tumors than in low-risk ones. Urinary bladder cancer is the fourth-most commonly diagnosed malignancy in men in the United States, accounting for 6.7% of all cancer cases [23]. Two-thirds to three-quarters of patients with bladder cancer are initially diagnosed as non-muscle-invasive tumors. The concept for treatment of non-muscle-invasive tumors is conservative. But some cases progress to muscle-invasive tumors after recurrence, which have a risk of metastasis and threating life. However, current molecular markers remain insufficient to predict the potential for tumor recurrence and progression precisely. PD-L1 expression is present on antigen-presenting cells (APCs), such as human monocytes, as well as activated human and murine dendritic cells [24]. *PD-L1* is a corregulatory ligand that can inhibit immune responses by either binding to PD-1 or a putative non-PD-1 receptor on the surface of T lymphocytes to induce antigen-specific T-cell apoptosis or anergy [24]. The role of PD-L1 was evaluated as a mechanism for local stage progression in cancer [24]. PD-L1 might act as a mediator of stage progression in bladder cancer. On the other hand, the association between PD-1/PD-L1 expression and progression is remains controversial. The correlation between PD-L1 expression in tumor cells and a worse clinical outcome was first reported in a study of 65 patients with bladder cancer by Nakanishi et al. [1]. Sharma et al. showed that the presence of PD-L1 tumor cells was not a predictor of prognosis [11]. Most reports have shown an association between the higher expression of PD-1/PD-L1 and a worse prognosis in bladder cancer.

Previous studies have implicated several cytokines, including IFNɤ, TNFα, and IL-2, as possible regulators of PD-L1 expression on the surface of several tumor cells [25–27], and IFNɤ has been thought as a strong inducer of PD-L1 expression in cancer cells [28]. Our study revealed that the expressions of STAT-1 and NFATc1 were positively correlated with PD-L1 expression. STAT-1 is downstream of IFNɤ and upstream of PD-L1 [29]. Following this cascade, NFAT is activated by PD-L1 [30]. We previously reported the anti-tumorigenic and oncogenic activity of NFAT in urothelial carcinoma and noted, in particular, that NFATc1 has a key role in its progression [16–18]. This correlation might therefore be a clue to reveal the anti-tumorigenic activity of PD-L1 through the STAT1-PD-L1-NFATc1 pathway.

The present study is associated with several limitations. First, this study was a retrospective analysis involving relatively few patients. Furthermore, all of the bladder cancer and paired normal tissue specimens were obtained via transurethral resection of the bladder, resulting in the potential for selection bias. Second, although the expression of *PD-1* and *PD-L1* differed between high-grade and low-grade cancers, we did not assess the therapeutic outcome or detailed mechanisms underlying the development of bladder cancer. Most studies involving PD-1 and PD-L1 antibodies are performed in the advanced stages of cancer and not as a primary therapy. Thus, further studies are needed, including central pathologists diagnosis. However, this is the first report in a Japanese cohort, which represents a strength of this study.

## Conclusions

*PD-1* and *PD-L1* might be new biomarkers that are correlated with the pathological grade of bladder cancer. *PD-L1* might act as a potential mediator of stage progression in bladder cancer. S*TAT1*-*NFAT* pathway might associate this role.

## Additional file

Additional file 1: **Figure S1.** The expression of *PD-L1* in human bladder cancer cell lines. (PPTX 71 kb)

## Abbreviations

NFAT: Nuclear factor of activated T-cells
PD-1: Programmed death 1
PD-L1: Programmed death ligand 1
qPCR: Quantitative real-time polymerase chain reaction
STAT 1: Signal transducer and activator of transcription 1
