# Supplementary figures and images for: PD-1 and PD-L1 are more highly expressed in high-grade bladder cancer than in low-grade cases: PD-L1 might function as a mediator of stage progression in bladder cancer
Source: BMC Urol. 2018 Nov 6;18:97. doi: 10.1186/s12894-018-0414-8 (PMC6219206; doi:10.1186/s12894-018-0414-8)

## Slide 1
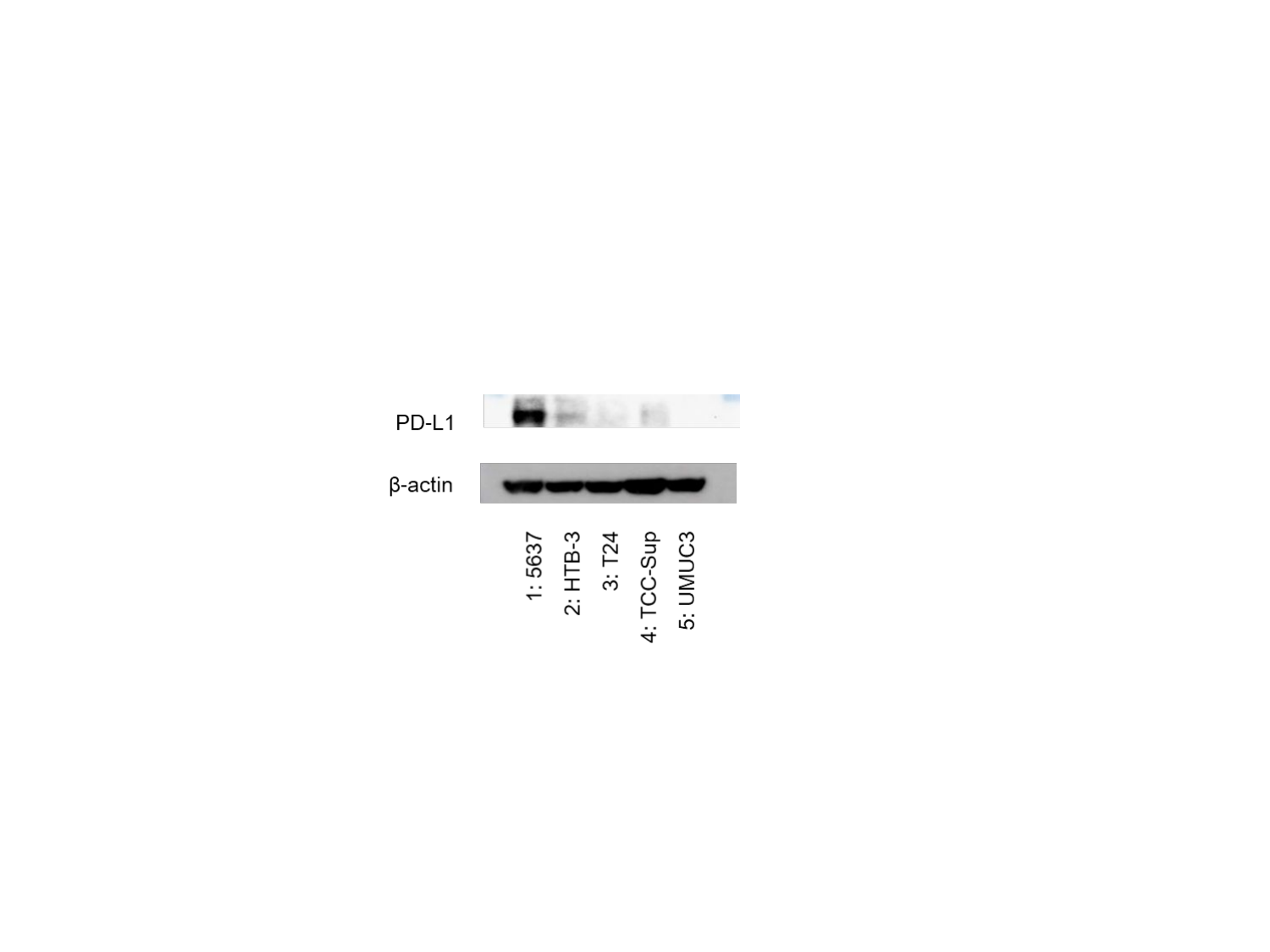

Supplement: Supplementary file 1 — Figure S1. The expression of PD-L1 in human bladder cancer cell lines. (PPTX 71 kb) [file 12894_2018_414_MOESM1_ESM.pptx]
